# Transcriptome and Gut Microbiota Profiling Revealed the Protective Effect of Tibetan Tea on Ulcerative Colitis in Mice

**DOI:** 10.3389/fmicb.2021.748594

**Published:** 2022-02-14

**Authors:** Ning Wang, Tao Wu, Di Du, Jie Mei, Huibo Luo, Zishan Liu, Muhammad Kashif Saleemi, Runhui Zhang, Candace Chang, Muhammad Aamer Mehmood, Hui Zhu

**Affiliations:** ^1^College of Bioengineering, Sichuan University of Science and Engineering, Zigong, China; ^2^School of Food and Biological Engineering, Xihua University, Chengdu, China; ^3^Process Technology Department, ExxonMobil Research and Engineering, Annandale, NJ, United States; ^4^Sichuan Jixiang Tea Co., Ltd., Ya’an, China; ^5^Department of Pathology, University of Agriculture, Faisalabad, Pakistan; ^6^Department of Veterinary Medicine, College of Animal & Veterinary Sciences, Southwest Minzu University, Chengdu, China; ^7^UCLA Microbiome Center, David Geffen School of Medicine at UCLA, Los Angeles, CA, United States; ^8^Department of Bioinformatics and Biotechnology, Government College University Faisalabad, Faisalabad, Pakistan

**Keywords:** ulcerative colitis, Tibetan tea, RNA-seq, gut microbiota, immune response

## Abstract

Traditionally, Ya’an Tibetan tea is routinely consumed by local people in the Tibet region. It is believed to possess promising anti-inflammatory benefits. This study was conducted to elucidate the protective impact of Tibetan tea extract (TTE) on dextran sodium sulfate (DSS)-induced colitis in mice. Mice were split into four groups: control (C) group, Tibetan tea (T) group, DSS-induced model (CD) group, and Tibetan tea + DSS (TD) group. The intake of TTE significantly reduced the clinical symptoms of ulcerative colitis (UC) by alleviating the impact of cellular damage and reducing glandular hypertrophy and the infiltration of inflammatory cells. UC led to a prominent shift of the microbial communities in the gut. Interestingly, the beneficial microbes, such as *Lactobacillus reuteri*, *Bifidobacterium choerinum*, and *Lactobacillus intestinalis*, were significantly increased in TTE-treated mice when compared to any other experimental group. The transcriptome analysis revealed that the positive effect of TTE on UC could be attributed to changes in the G alpha (i) signaling pathway and the innate immune system. The genes related to inflammation and immune system pathways were differentially expressed in the TTE-treated group. Moreover, the relative expression of genes linked to the inflammatory TLR4/MyD88/NF-κB signaling pathway was significantly downregulated toward the level of normal control samples in the TD group. Overall, this study revealed the modulatory effect by which TTE reversed the development and severity of chronic colon damage.

## Introduction

Ulcerative colitis (UC) can trigger hemorrhagic diarrhea and passage of mucus and/or pus, which causes cramping in the abdominal during bowel movements (Ananthakrishnan et al., 2018). Due to the prolonged disease duration and recurrent attacks, the World Health Organization (WHO) has listed it among the modern refractory diseases (Caruso et al., 2020). It is believed that excessive inflammation of the intestine occurs due to an imbalance in the gut microbiota and immunity of the mucosa, resulting in UC (Limdi and Vasant, 2015; Ajayi et al., 2018). Currently, the available drugs are inefficient in the remediation of the disease and cause several side effects. It is therefore essential to explore safer, more effective, and cost-effective alternatives to prevent dysbiosis (Oh et al., 2020).

Tibetans are at high risk of suffering from UC due to their extreme living environment and eating habits, being on a plateau, making it necessary for them to drink tea daily for its health benefits (Lewis and Abreu, 2017). Tibetan tea is a product of geographical indication exclusively produced in the Ya’an region in China. It is a type of black tea made from a mature small-leaf cultivar, which is picked from high up the mountains above 1,000 m (Zheng et al., 2020). Pile fermentation is the key process to achieving the characteristic features of Tibetan tea (Xu et al., 2020).

Microbial dysbiosis is among the major pathological factors of UC that affects host mucosal immunity (Franzosa et al., 2019; Liu et al., 2020; Lee and Chang, 2021). Pathogenic bacteria, along with opportunistic pathogens, interact with the gut mucosa either directly or indirectly by secreting toxins, which cause an improper mucosal immune response in the intestine (Qi et al., 2018). The dysregulated immune system responds in the form of inflammation, leading to the development of UC. Multiple immune cells, including T lymphocytes, macrophages, and neutrophils, are abnormally activated and do not only elicit acute colitis but also further damage the colon by upregulating the pro-inflammatory cytokines such as tumor necrosis factor alpha (TNF-α), interleukin 1 beta (IL-1β), and IL-6 (Sun et al., 2020). Thus, regulating the gut microbiota may be a promising strategy to preventing and/or treating UC (Petrella, 2016; Lee et al., 2018; Sun et al., 2018).

Based on the long-term consumption of Tibetan tea in the daily life of Tibetans, it was hypothesized that Tibetan tea polyphenols have preventive effects against UC. Accordingly, the current study evaluated the composition and anti-inflammatory effects of Tibetan tea extract (TTE) on dextran sodium sulfate (DSS)-induced colitis in mice. The impact of TTE on the expression of colonic genes and on the gut microbiota was elucidated using transcriptome combined with DNA sequencing analysis. These data were employed to identify the altered metabolic and immune pathways, as well as the key microbiota regulatory “hubs,” that help elucidate the novel insights into the anti-inflammatory effects of TTE.

## Materials and Methods

### Preparation of Tibetan Tea Extract

The Tibetan tea (*Camellia sinensis*) used in this study was of the first class and provided by Sichuan Jixiang Tea Co., Ltd. (Ya’an, China). It was made from tea with one bud and one leaf as raw materials and had been stored in a dry and ventilated warehouse at 24–28°C for 1 year. One hundred grams of Tibetan tea was pulverized and passed through an 80-mesh sieve. The powder was decocted in 50-fold volume using boiling water for 10 min. The supernatant was collected by centrifugation at 5,000 rpm for 10 min and was concentrated under reduced pressure using a rotary evaporator. TTE was lyophilized under 10 Pa at –55°C and stored at 4°C for further use.

### Determination of the Major Compounds in Tibetan Tea

The total phenolic content (TPC) of the TTE samples was quantified using the Folin–Ciocalteu colorimetric assay. The results were expressed as milligrams of gallic acid equivalent (GAE) per 100 mg of dried extract. The total flavonoid content (TFC) was determined using the colorimetric method, and the results were expressed in milligrams rutin equivalent (RE) per 100 mg dry weight (Wu et al., 2016). The composition of Tibetan tea was evaluated with untargeted metabolomics as described previously (Li et al., 2021).

### Ethics Statement

Mice were purchased from Vital River Laboratory Animal Technology Co. Ltd. (Beijing, China). All experiments were executed following the Guide for the Care and Use of Laboratory Animals by Sichuan University of Science and Engineering (Zigong, China), after seeking proper approval.

### Experimental Setup to Induce UC in Mice

**FIGURE 1:**
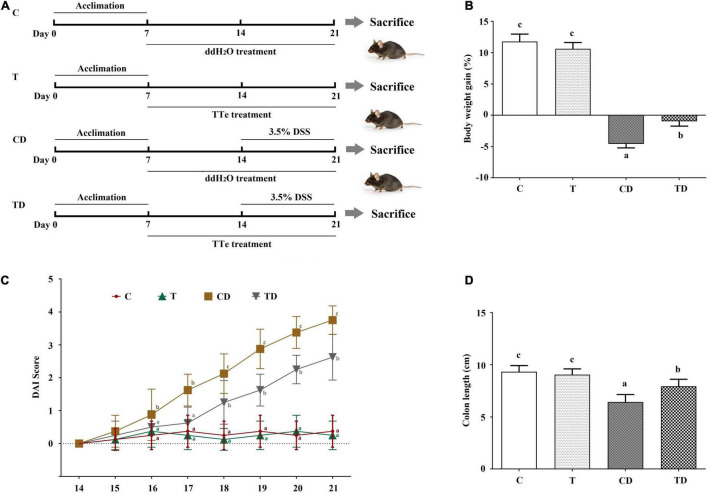
Effect of Tibetan tea on the colon tissues of dextran sodium sulfate (DSS)-induced ulcerative colitis (UC) in mice. **(A)** Schema of the experimental design. **(B)** Body weight gain. **(C)** Disease Activity Index (DAI) scores during DSS treatment. **(D)** Colon length. Data were expressed as the mean ± SD. *Lowercase letters a*–*c* indicate significant differences in all treatment groups (*p* < 0.05). *C*, control group (*n* = 8); *T*, Tibetan tea group (*n* = 8); *CD*, DSS model group (*n* = 8); *TD*, Tibetan tea + DSS group (*n* = 8).

C57BL/6J mice (male, 18–22 g) were kept at 23 ± 1°C under a 12-h light/dark cycle. Unlimited standard rodent diet and water were provided. After 1 week, 32 mice were indiscriminately split into four experimental groups: the control (C) group, the Tibetan tea (T) group, the DSS model (CD) group, and the Tibetan tea + DSS (TD) group. Mice in the T and TD groups were orally administered 100 mg of TTE per kilogram body weight, while mice in the C and CD groups were treated with the same volume of ddH_2_O (Figure 1A). After pretreatment for 7 days, the mice in the CD and TD groups were administered 3.5% DSS (MP Biochemicals, Irvine, CA, United States) in drinking water given *ad libitum* (days 8–14), as described (Fang et al., 2019). Body weight, stool blood, and the consistency of the stool (to calculate the Disease Activity Index, DAI) were recorded three times a day during the duration of the experiments (Jeengar et al., 2017).

Mice were sacrificed on day 21 (Figure 1A), and blood and cecum samples were collected for further study. The colons, taken from the ileocecal junction to the anal verge, were dissected and measured for weight and length before being longitudinally opened and washed using phosphate-buffered saline (PBS) at pH 7.4. Part of the colon tissues was fixed in 10% formalin (neutral-buffered) for subsequent histological analyses. The remaining colon samples were stored in liquid nitrogen until further use.

### Histopathological Assays

The transverse rings of the colons were fixed in 4% buffered formalin and embedded in paraffin before staining with hematoxylin and eosin (H&E) (Leica Biosystems, Wetzlar, Germany). Goblet cells were observed with Alcian blue/periodic acid–Schiff (AB/PAS) straining. These sections were observed under a microscope (BX53M; Olympus, Tokyo, Japan) and were scored based on a four-point scale (0–4) upon examination for exudates, polymorphonuclear leukocyte invasions, epithelial damage, submucosal edema, and necrosis (Kuprys et al., 2020; Ma et al., 2020).

### Immunohistochemical Analyses

Paraffin-embedded sections of the colonic tissues (5 mm thickness) were prepared and examined using mouse anti-myeloperoxidase (MPO) antibodies (NOVUS Biologicals, Littleton, CO, United States) as described previously (Lee et al., 2018). The positive rate of MPO cells (the number of positive cells/total cell number × 100) was calculated using HALO (Indica Labs, Albuquerque, NM, United States).

### DNA Sequencing

Total DNA was extracted from all cecal digesta samples (75 mg sample was used) using the cetyltrimethyl ammonium bromide/sodium dodecyl sulfate (CTAB/SDS) method (Griffith et al., 2009). The quality and the quantity of the DNA were assessed using gel electrophoresis (1% agarose gel) and a NanoDrop Spectrophotometer (ND-1000; Thermo Fischer Scientific, Waltham, MA, United States), respectively. DNA was used as the template to amplify the V4 hypervariable region of the 16S ribosomal RNA (rRNA) gene and sequenced by Novogene Bioinformatics Technology Co. Ltd. (Beijing, China).

### Sequence Analyses

Single-end reads were cleaned by removing the primer sequences and were subjected to quality filtration following the recommended quality control parameters in cutadapt V1.9.1 (Martin, 2011). Chimera sequences were removed using the SILVA reference database and the UCHIME algorithm (Edgar et al., 2011; Haas et al., 2011). The cleaned reads were analyzed by Uparse v7.0.1001, and sequences with ≥97% similarity were grouped into the same operational taxonomic units (OTUs). The SILVA database and the Mothur algorithm were employed for taxonomy assignment of the representative sequences of each OTU (Edgar, 2013). Finally, multiple sequence alignments were performed using MUSCLE v.3.8.31 (Edgar, 2004). Alpha diversity and Jackknifed beta diversity analyses were performed as described previously (Schloss et al., 2009).

### RNA Isolation for Library Construction

To further investigate the molecular mechanism of Tibetan tea in preventing DSS-induced UC in mice, colon samples were separately collected from the C, CD, and TD groups, and total RNA was extracted using the TriZol reagent as described by the manufacturer (Invitrogen, Carlsbad, CA, United States). After determining the RNA quality and quantity, the libraries were constructed and sequenced on the Illumina HiSeq X Ten platform. Subsequently, 150-bp paired-end reads were obtained.

### RNA-Seq Analyses

The assembly and functional assignment of the RNA sequencing (RNA-seq) data were executed following a method described previously (Pandurangan et al., 2015). Two groups/conditions (in duplicate) were subjected to differential expression analyses using the DESeq2 R package (1.16.1). Gene Ontology (GO) analysis of differentially expressed genes (DEGs) was performed using the clusterProfiler R package, and gene length bias was corrected for. GO terms with corrected *p* < 0.05 were considered as enriched by DEGs. The statistical enrichment of DEGs in the Kyoto Encyclopedia of Genes and Genomes (KEGG) pathways was examined through the clusterProfiler R package.

Deseq2 (PMID: 25516281) was used to normalize the read counts in order to enable cross-sample comparisons. Accordingly, 2,526 genes showed differential expression between the CD and C groups, and 337 showed differential expression between the TD and CD groups.

### Pathway Analyses

Pathway analyses were performed using the Reactome and KEGG databases. Protein–protein interactions were analyzed using STRING (PMID: 30476243) and were colored by the corresponding Reactome pathways. A KEGG gene–pathway clustered heatmap was calculated using Enrichr (PMID: 23586463 and PMID:27141961).

### Real-Time Quantitative PCR

To further validate the key genes and pathways involved in the anti-inflammatory effects of TTE, real-time quantitative PCR (RT-qPCR) was performed to detect the relative expression levels of *IL-1*β, *IL-6*, *IL-10*, *MyD88*, *NF-*κ*B*, *TLR4*, and *TNF-*α using the 2^–ΔΔCt^ method, while β*-actin* expression was used as the reference (Supplementary Table 1). The high-quality RNA extracted from the colon tissues was used as a template to synthesize complementary DNA (cDNA).

### Statistical Analyses

All experiments were repeated three times, and data were shown as the mean ± SD. One-way ANOVA with Tukey’s *post hoc* test was employed using GraphPad Prism 6.0 (San Diego, CA, United States). Data with *p*-values < 0.05 were considered significant.

## Results

### Characterization of the Major Ingredients of Tibetan Tea

Tea polyphenols and flavonoids could be the major components responsible for the health benefits of TTE. Therefore, the contents of total polyphenols and total flavonoids in TTE were evaluated with spectroscopy, which were 70.24 ± 3.12 mg GAE/g and 104.57 ± 7.61 mg RE/g, respectively. Furthermore, the MS/MS fragment ions of TTE were analyzed using ultra-high-performance liquid chromatography/time-of-flight tandem mass spectrometry (UHPLC-TOF-MS/MS) (Supplementary Table 2). A total of 17 compounds were tentatively identified using MS-DIAL 4.6 software^1^, including phenolic acids, flavonoids, and theaflavins. It is worth noting that the content of theaflavins in TTE was 3.3 times higher than that in Pu-erh tea (Supplementary Figure 1).

### Tibetan Tea Extract Cured Dextran Sodium Sulfate-Induced Ulcerative Colitis

The DAI is a conventional index for evaluating colitis severity. Here, the DAI of the T group was little changed when compared to the DAI scores of mice in the C group. Administration of DSS resulted in apparent clinical symptoms such as diarrhea, weight loss, and fecal blood in mice of the CD and TD groups (Figure 1B). Accordingly, a significant increase in DAI was observed in the CD group on day 17 when compared to the control, and the maximum DAI was reached on day 21 (3.750 ± 0.433) (Figure 1C). Delayed and/or reduced symptoms, including reduced fecal occult blood and diarrhea, in response to TTE treatment were observed. The DAI of the TD group considerably decreased. These data demonstrated the protective effects of Tibetan tea against murine ulcerative colitis (Figure 1C). DSS administration also shortened the colonic length of mice from the CD group (6.43 ± 0.72 cm) when compared to mice in the C group (9.34 ± 0.59 cm, *p* < 0.05) (Figure 1D and Supplementary Figure 2), and TTE treatment was preventive against the shortening of the colon (7.94 ± 0.68 cm, *p* < 0.05) in DSS-administered mice. No of the experimental mice died during the experimental period.

### Histopathology of the Colon and Calculation of the MPO-Positive Rate

**FIGURE 2:**
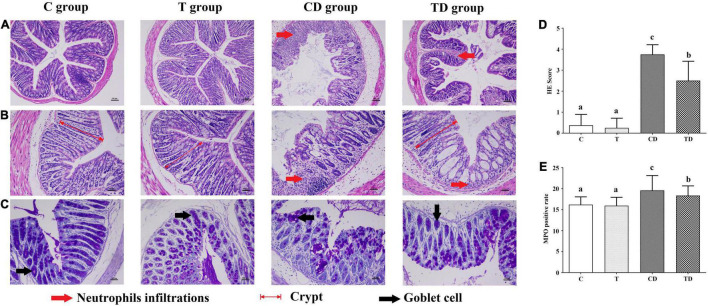
Histopathological analysis of colon segments. **(A,B)** Staining of colon tissues of mice with hematoxylin–eosin (H&E), original magnifications of × 100 **(A)** and × 200 **(B)**. **(C)** Goblet cells observed with Alcian blue/periodic acid–Schiff (AB/PAS) staining. **(D)** Determination of the histological scores for the control (C), dextran sodium sulfate (DSS) model (CD), and Tibetan tea + DSS (TD) groups. **(E)** Sections immunostained with mouse anti-myeloperoxidase (MPO) antibody in each group, with the positive cells staining brown. The positive rate of MPO cells (the number of positive cells/total cell number × 100) was calculated using HALO. *Lowercase letters a*–*c* indicate significant differences in all treatment groups (*p* < 0.05).

Histopathological evaluation of the colons demonstrated that the TTE group also showed protective effects against DSS-induced colitis (Figure 2). Both the C and T groups showed normal colonic mucosa with the epithelium, crypts, and the submucosa intact. However, in the colons of the CD group, loss of epithelial and goblet cells along with severe neutrophil infiltration, deformation of the crypt structure, appearance of crypt abscesses, and ulcers were observed. Interestingly, mice treated with TTE showed alleviation of DSS-induced hemorrhage in the colon. After 7 days of DSS administration, the histological scores were 3.75 ± 0.46 in the CD group and 2.50 ± 0.93 in the TD group (Figure 2D). In addition, the results of MPO protein immunohistochemistry showed that the MPO content in the colon tissues of mice in the CD group was substantially higher compared to that in the C group, but significantly decreased in the TD group (Figure 2E).

### Tibetan Tea Extract Altered the Cecal Microbiota in Dextran Sodium Sulfate-Induced UC Mice

The IonS5TMXL platform was used to extract the single-end sequences of 16S rRNA genes, where a total of 1,844,712 high-quality reads remained, with an average of 76,863 reads per sample after deleting chimeras and low-quality reads. The overall effective rate of the quality control was 94.45%. These sequences were assigned to 875 OTUs based on similarity (≥97%). Among these, 440 (50.29%) OTUs were assigned to the genus level.

**FIGURE 3:**
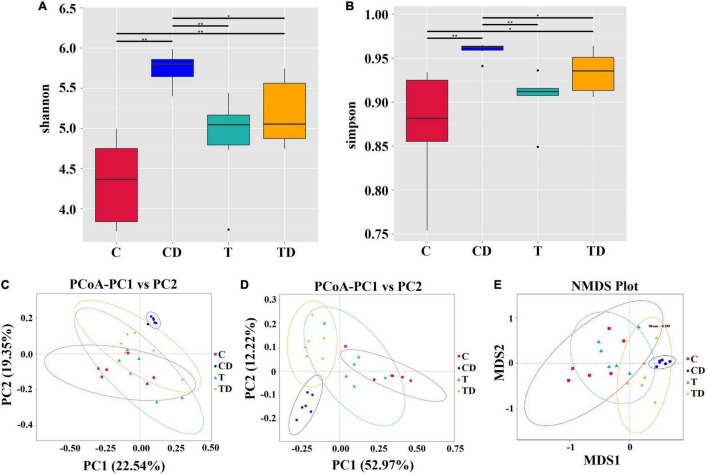
Differences in the cecal microbiota of mice. **(A,B)** The diversity and richness of the microbiota of mice in different groups were measured by the Shannon **(A)** and Simpson **(B)** indices. *Asterisk above the box plots* indicates significant differences between age groups (Wilcoxon rank-sum test: *p < 0.05, **p < 0.01). **(C,D)** Principal coordinates analysis (PCoA) of microbial communities based on weighted **(C)** and unweighted **(D)** Unifrac distances. **(E)** Non-metric multidimensional scaling analysis.

**FIGURE 4:**
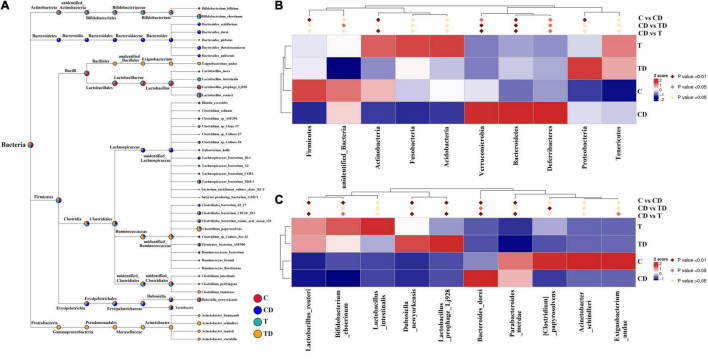
Comparison of the microbial communities in mice. **(A)** Evolutionary tree of the top 100 genera. **(B)** Microbial taxa differentially distributed between groups at the phylum level. **(C)** Identification at the species level by MetaStat analysis.

Comparison of the alpha diversity between the CD and TD groups showed that the Shannon and Simpson indices of the TD group were substantially lower than those of the CD group (Wilcoxon rank-sum test: *p* < 0.05) (Figures 3A,B). Obvious clustering was observed within the treatment groups in the principal coordinates analysis (PCoA) plot based on weighted and unweighted Unifrac and in the non-metric multidimensional scaling (NMDS) plot based on Bray–Curtis distances, suggesting that the CD and TD groups were composed of distinct bacterial communities (Figures 3C–E). Analysis of similarities (ANOSIM) also proved that differences in the community membership between both groups were statistically significant (*r* = 0.6907, *p* = 0.001). MetaStat was also used to look for bacterial taxa that show substantial differences in abundance (Figure 4). At the phylum level, the relative abundances of Bacteroidetes, Verrucomicrobia, Deferribacteres, and Melainabacteria decreased significantly (*p* < 0.05) in the TD group in comparison to the CD group. At the species level, the relative abundance of *Bacteroides dorei* decreased significantly (*p* < 0.05), while that of *Clostridium papyrosolvens* increased significantly (*p* < 0.01) in the TD group compared to the CD group. Comparison of the cecal microbiota of mice in the four groups revealed that beneficial microbes such as *Lactobacillus reuteri*, *Bifidobacterium choerinum*, and *Lactobacillus intestinalis* showed an “up–down–up” trend across the C, T, CD, and TD groups (Figure 4C).

The microbial community compositions of mice are shown in Figure 4A. MetaStat analysis was used to identify the differential distribution of the microbial taxa between groups at the phylum and species levels (Figures 4B,C). The relative abundances of the phyla Actinobacteria and Firmicutes in the CD group decreased significantly compared to those in the control group (*p* < 0.01), while those of the Proteobacteria, Bacteroidetes, Verrucomicrobia, and Deferribacteres increased significantly (*p* < 0.05). At the species level (top 10), the relative abundances of *L. reuteri*, *B. choerinum*, *Lactobacillus prophage* Lj928, *Dubosiella_newyorkensis*, and *Exiguobacterium undae* significantly reduced in the CD group compared to those in the control group (*p* < 0.05), whereas the relative abundances of *Parabacteroides merdae* and *Bacteroides dorei* significantly increased in the CD group (*p* < 0.05).

### Transcriptome Data Analysis

**FIGURE 5:**
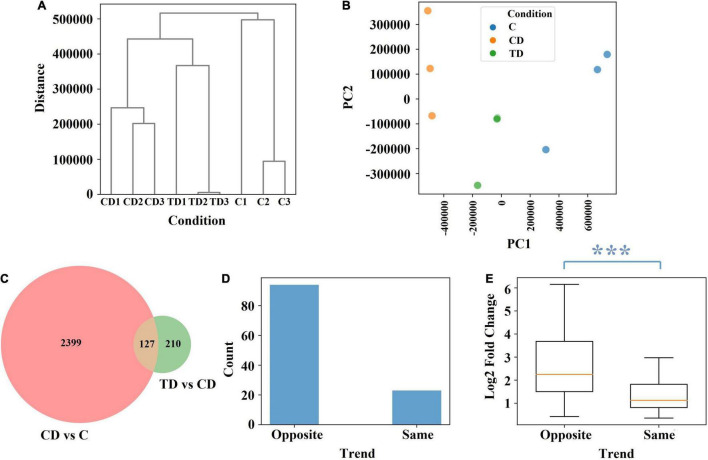
The effect of Tibetan tea at the genetic level. **(A)** Hierarchical clustering dendrogram of normalized gene expression. **(B)** Principal component analysis of normalized gene expression. **(C)** Venn diagram of the differential expression between CD *vs*. C and between TD *vs*. CD. **(D)** Trend count of 127 common genes. **(E)** Log2 fold change of the 127 common genes. The difference between the two trends is characterized using the Mann–Whitney *U* test *p*-value. ***p < 0.005. *TD*, Tibetan tea + DSS; *CD*, dextran sodium sulfate-induced model; *C*, control.

To further elucidate the protective effects seen in TTE-treated mice, the colonic tissues of mice were subjected to transcriptomic analysis. Accordingly, a total of 1,224,984,344 raw sequence reads were generated from the colonic tissues of mice, and 1,172,856,536 clean reads were retained after filtering (Supplementary Table 3). The RNA-seq data were subjected to an unsupervised hierarchical clustering and principal component analysis (PCA; Figures 5A,B). The fact that all samples within each group were clustered together also validated the robustness of the data collection process. In the PCA model, the top 2 components explained 79 and 16% of the variance, respectively. After TTE treatment, the genetic profile in the TD group was shown to be closer to that of the C group, which confirmed that Tibetan tea had a positive effect on the induced UC at the genetic level.

Further comparisons of the different groups showed that 2,526 genes were differentially expressed between the CD and C groups and 337 genes between the TD and CD groups, among which 127 genes were common (Figure 5C). Among the 127 common genes, 94 showed an opposite trend across the C, CD, and TD groups, namely, upregulated at CD *vs*. C and downregulated at TD *vs*. CD or downregulated at CD *vs*. C and upregulated at TD *vs*. CD (Figure 5D). This finding verified that Tibetan tea is effective at reducing inflammation by regulating genes toward the level of normal control samples. It is worth noting that the 94 genes that showed the opposite trend across the C, CD, and TD groups also had significantly higher fold change values (Figure 5E).

### Pathways Responsive to Tibetan Tea Extract Treatment

**FIGURE 6:**
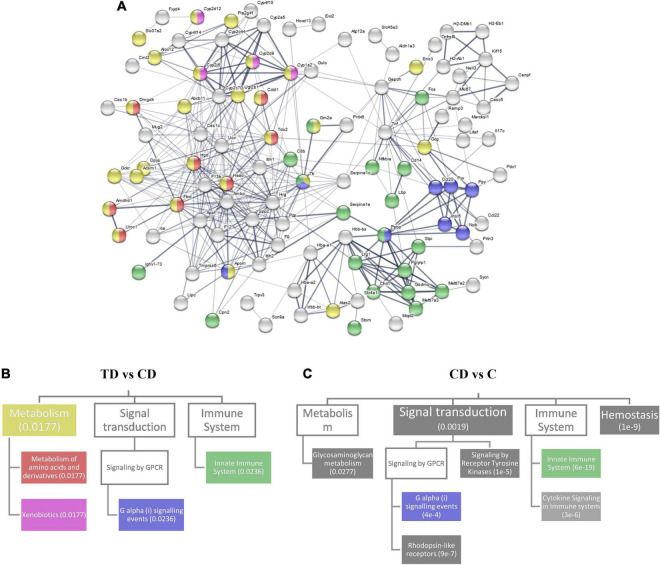
Reactome pathway analysis. **(A)** Protein–protein interaction network for differential gene expression between TD and CD. **(B)** Top 20 related Reactome pathways for differential expression in TD and CD. The pathways are mapped in **(A)** with corresponding colors. **(C)** Top 20 related Reactome pathways for differential expression in CD and C. *Numbers within parentheses* represent the false discovery rate. *Boxes with no color* represent a false discovery rate >0.05. *TD*, Tibetan tea + DSS; *CD*, dextran sodium sulfate-induced model; *C*, control.

The protein–protein interactions of the DEGs between the TD and CD groups were analyzed and visualized in a network fashion (Figure 6A). The thickness of the edges represents the confidence of data support. Here, three Reactome pathways were identified—metabolism, G alpha (i) signaling events, and innate immune system—which covered most of the interaction networks with high-confidence edges. The relationship between these three pathways and their associated pathways that were within the top 20 pathways ranked by the false discovery rate are shown in Figure 6B. Two associated pathways of metabolism—amino acid metabolism and xenobiotics—were shown to be related to the differential expression between the TD and CD groups. The same analysis for the differential expression between CD and C (Figure 6C) was also performed. Several pathways, including signal transduction, innate immune system, hemostasis, and glycosaminoglycan metabolism, covered most of the interaction network with high-confidence edges between the CD and C groups. A comparison between TD *vs*. CD and CD *vs*. C showed that the positive effect of TTE in controlling UC could be attributed to the changes in the G alpha (i) signaling pathway and the innate immune system. The fact that the xenobiotics pathway was highly differentially regulated in the TD *vs*. CD comparison, but not in the CD *vs*. C comparison, also confirmed that the changes in the pathways were due to exogenous compounds.

**FIGURE 7:**
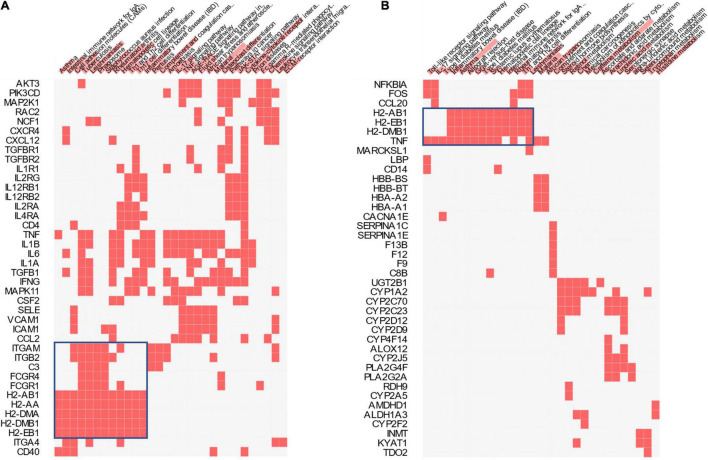
Kyoto Encyclopedia of Genes and Genomes (KEGG) pathway analysis. Clustered heatmap of the KEGG genes and pathways for CD *vs*. C **(A)** and TD *vs*. CD **(B)**. *Color bars over the pathway names* represent the combined scores (*p*-values and *z*-scores) of the pathways. *TD*, Tibetan tea + DSS; *CD*, dextran sodium sulfate-induced model; *C*, control.

In addition to the Reactome pathways, the KEGG pathways associated with the differential expression between the TD and CD groups and those associated with the differential expression between the CD and C groups were also compared (Figure 7). The largest cluster in each comparison (boxed in blue) corresponding to several inflammation- and immune system-related pathways, including the intestinal immune network, Toll-like receptor signaling pathway, and IL-17 signaling pathway, were identified.

### Gene Expression Involved in the TLR4/MyD88/NF-κB Signaling Pathway

**FIGURE 8:**
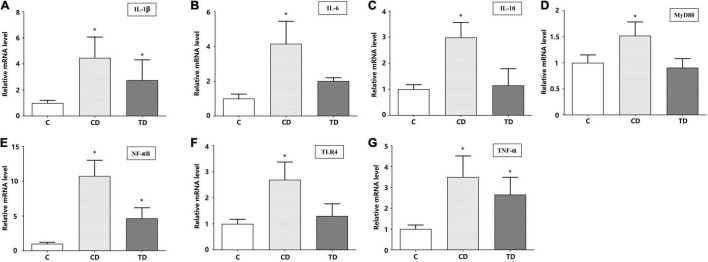
The relative mRNA expression levels of IL-1β **(A)**, IL-6 **(B)**, IL-10 **(C)**, MyD88 **(D)**, NF-κB **(E)**, TLR4 **(F)**, and TNF-α **(G)** in colon tissues. *p < 0.05 vs. the control group.

We measured the relative mRNA expressions of the TLR4/MyD88/NF-κB signaling pathway-related genes (*IL-1*β, *IL-6*, *IL-10*, *MyD88*, *NF-*κ*B*, *TLR4*, and *TNF-*α) in the colons. DSS administration significantly elevated the mRNA expressions of *IL-1*β, *IL-6*, *IL-10*, *MyD88*, *NF-*κ*B*, *TLR4*, and *TNF-*α up to 4. 46–, 4. 16–, 2. 99–, 1. 52–, 10. 76–, 2. 69–, and 3.50-fold, respectively, in DSS-induced mice compared to mice in the C group (*p* < 0.01). The mRNA levels in mice with TTE pretreatment (TD group) were downregulated to nearly normal levels (*p* > 0.05) (Figure 8). These data confirmed that the anti-inflammatory impact of beneficial microbes correlated with the downregulation of the TLR4/MyD88/NF-κB signaling pathway.

## Discussion

Under muggy conditions with an abundance of environmental microorganisms, tea polyphenols go through oxidation, condensation, and hydrolysis, making Tibetan tea rich in a variety of phenolics, theaflavins, and thearubigins, among others. Pu-erh tea, a well-known black tea, is made from a large-leaf cultivar, whereas Tibetan tea is produced from local small-leaf cultivar. As a result, TTE contains high levels of theaflavins than Pu-erh tea. It was reported for the first time that the preventive effects of TTE on DSS-induced UC in mice were further explored comprehensively using transcriptome and DNA sequencing analyses.

In the current study, TTE intake significantly reduced the clinical symptoms of UC by alleviating the morphology of damaged cells and reducing glandular hypertrophy and the infiltration of inflammatory cells. An increase in the concentration of MPO is regarded as an index of the infiltration of neutrophils and inflammation (Choudhary et al., 2001). Herein, TTE significantly decreased the concentration of MPO when compared to non-treated DSS-administered mice.

There are two potential mechanisms of TTE preventing UC. The first mechanism is the indirect regulation of the gut microbiota. Pathogens damage or weaken the protective function of the intestinal mucosa, while beneficial microbes can counteract and/or fix such damage (Lee and Chang, 2021). When mice were treated with TTE, the relative abundance of Bacteroidetes, Verrucomicrobia, Deferribacteres, and Melainabacteria decreased significantly at the phylum level. These results indicated that TTE could alleviate UC by regulating the number of pathogenic bacteria in the gut microbiota. In the C, T, CD, and TD groups, beneficial microbes, such as *L. reuteri*, *B. choerinum*, and *L. intestinalis*, showed an “up–down–up” trend. It was demonstrated that TTE could significantly upregulate *Lactobacillus* and *Bifidobacterium* both in healthy and UC-induced mice and that these beneficial microbes were closely associated with colitis remission.

*Bifidobacterium* is one of the first beneficial microbes to colonize the intestines in babies and remains the major microbe of the intestines in adults (Ventura et al., 2014). It increases antioxidant activity and impedes the recurrence of UC. *Bifidobacterium breve* has been shown to reduce the apoptotic loss of epithelial cells in a MyD88-dependent fashion (Hughes et al., 2017). *Lactobacillus*, another beneficial commensal, has been shown to adjust the community of the gut microbiota along with inhibition of harmful bacteria *via* lowering the gut pH, producing short-chain fatty acids (SCFAs) and defensins (Durchschein et al., 2016). In addition, *Lactobacillus* prevents the colonization of harmful bacteria and the invasion of colon tissues and decreases the pro-inflammatory response of epithelial cells (Takamura et al., 2011). The probiotic *L. reuteri* produces reuterin, which contains hydroxy and aldehydic functional groups, and has been shown to have a robust repressive effect on intestinal pathogens (Takamura et al., 2011; Sun et al., 2018). Thus, it was expected that Tibetan tea could alleviate inflammatory reactions and prevent UC by increasing these beneficial microbes in the gut.

The second mechanism is the direct regulation of the host intestinal immune system to control inflammation. To explore the molecular mechanism of the protective effects of TTE on UC and to identify the key gene sets and pathways involved in this anti-inflammatory process, we focused on detecting changes in the C, CD, and TD groups. The colonic tissues of the mice from these three groups were subjected to transcriptomic and qPCR analyses. By analyzing the transcriptome, the positive effect of TTE on UC is likely attributed to the changes in the G alpha (i) signaling pathway and the innate immune system. The DEGs between the CD and TD groups were enriched in several inflammation- and immune system-related pathways (intestinal immune network, Toll-like receptor signaling pathway, IL-17 signaling pathway, etc.). The pathogenesis of inflammatory bowel disease (IBD) has been linked to an imbalanced and overactive immune response of the intestinal mucosa (Ramos and Papadakis, 2019). DSS damages the intestinal epithelial barrier; as a result, pathogens cross the mucosa and infect the epithelial cells, subsequently causing inflammation (Jeengar et al., 2017).

NF-κB, a key transcription factor, has been shown to be activated in the colon of IBD patients, where its activation level was shown to be correlated with disease severity (Meng et al., 2020). Therefore, modulating the NF-κB pathway could be an effective strategy to treat IBD. Interestingly, the positive impact of TTE on UC was attributed to changes in the innate immune system. Toll-like receptors (TLRs) elicit inflammatory reactions by triggering the inherent immune system (Alfonso-Loeches et al., 2010). For instance, TLR4 normalizes several inflammatory cytokines in IBD (Furuta et al., 2006). Furthermore, the relative expression of the genes linked to the TLR4/MyD88/NF-κB signaling pathway (*IL-1*β, *IL-6*, *IL-10*, *MyD88*, *NF-*κ*B*, *TLR4*, and *TNF-*α) was verified in the colons of mice. The results clearly suggest that the anti-inflammatory impact of beneficial microbes is linked to the decreased expression of the TLR4/MyD88/NF-κB signaling pathway in the colonic tissues of mice.

## Conclusion

Our data provided orthogonal evidence to confirm that TTE can regulate the immune system to reduce inflammation in UC. This was attributed to the effective regulation of the inflammatory signaling pathway (TLR4/MyD88/NF-κB) and the recovery of beneficial microbes belonging to the genera *Lactobacillus* and *Bifidobacterium*. This study advances the understanding of the protective effects of Tibetan tea in alleviating enteritis. Moreover, future investigations should focus on exploring the components of TTE that conferred the main anti-inflammatory impact.

## Data Availability Statement

The raw sequence data reported in this paper have been deposited in the Genome Sequence Archive (Genomics, Proteomics & Bioinformatics 2017) in National Genomics Data Center (Nucleic Acids Res 2021), China National Center for Bioinformation/Beijing Institute of Genomics, Chinese Academy of Sciences, under accession number CRA004155 and CRA004148 that are publicly accessible at https://bigd.big.ac.cn/gsa.

## Ethics Statement

The animal study was reviewed and approved by the Care and Use of Laboratory Animals by Sichuan University of Science and Engineering (Zigong, China).

## Author Contributions

NW and HZ conceived the study. NW and DD designed the study and wrote the first version of the manuscript. TW, HL, and ZL participated in its design and coordination and performed the statistical analysis. JM and RZ conceived the study and collected the experimental material. MS and MM collected and analyzed the raw data. MM revised the manuscript. HZ is responsible for this study, participated in its design and coordination, and helped draft the manuscript. All authors have read and approved the final manuscript.

## Conflict of Interest

DD was employed by the company ExxonMobil Research and Engineering. JM was employed by Sichuan Jixiang Tea Co., Ltd. The remaining authors declare that the research was conducted in the absence of any commercial or financial relationships that could be construed as a potential conflict of interest.

## Publisher’s Note

All claims expressed in this article are solely those of the authors and do not necessarily represent those of their affiliated organizations, or those of the publisher, the editors and the reviewers. Any product that may be evaluated in this article, or claim that may be made by its manufacturer, is not guaranteed or endorsed by the publisher.

## Footnotes

1
http://prime.psc.riken.jp/compms/msdial/main.html

## Supplementary Material

Supplementary Figure 1 Comparison of theaflavins content between Tibetan tea **(A)** and Pu-erh tea **(B)** by UHPLC-Q-TOF.

Supplementary Figure 2 Effects of TTE on colon length of mice after colitis induction. The arrow represents the site of colonic edema. Scale bars = 1 cm.
