# Supplementary material for: Transcriptome and Gut Microbiota Profiling Revealed the Protective Effect of Tibetan Tea on Ulcerative Colitis in Mice
Source: Front Microbiol. 2022 Feb 14;12:748594. doi: 10.3389/fmicb.2021.748594 (PMC8882814; doi:10.3389/fmicb.2021.748594)
Supplement: Supplementary file 1 [file Data_Sheet_1.docx]

**Table S.1 Primers sequences for Real-Time Quantitative PCR**

| **Gene** | **Full name** | **Primers** | |
| --- | --- | --- | --- |
|  |  | **Forward Sequence** | **Reverse Sequence** |
| IL-1β | Interleukin 1 beta | CTGAACTCAACTGTGAAATGC | TGATGTGCTGCTGCGAGA |
| IL-6 | Interleukin 6 | GAGGATACCACTCCCAACAGACC | AAGTGCATCATCGTTGTTCATACA |
| IL-10 | Interleukin 10 | ACAGCCGGGAAGACAATAAC | CAGCTGGTCCTTTGTTTGAAAG |
| MyD88 | Myeloid differentiation primary response gene 88 | GCATGGTGGTGGTTGTTTCTG | GAATCAGTCGCTTCTGTTGG |
| NF-κB | Nuclear factor of kappa B | ACACTGGAAGCACGGATGAC | TGTCTGTGAGTTGCCGGTCT |
| TLR4 | Toll-like receptor 4 | ATCGCCTATGGTTGTTGACC | GGTTTCACGACTGGAGGTTC |
| TNF-α | Tumor necrosis factor alpha | AGGGTCTGGGCCATAGAACT | CCACCACGCTCTTCTGTCTAC |
| *β*-actin | Beta actin | AATCGTGCGTGACATCAA | GCTCGTTGCCAATAGTGA |

**Table S.2** **Summary of** **tentatively identified compounds from Tibetan tea by UHPLC-Q-TOF**

| **No.** | **precursor ion** | **Molecular**  **formula** | **Characteristic Fragment ions** | **Tentatively Identification** |
| --- | --- | --- | --- | --- |
|  |  |  | **Negative mode** |  |
| 1 | 191.15585 | C_7_H_12_O_6_ | 127.0043;85.005;58.9961 | D-(-)-quinic acid |
| 2 | 134.02048 | C_5_H_5_N_5_ | 120.0986;107.0049;89.7836;55.167 | Adenine |
| 3 | 305.00519 | C_15_H _14_O_7_ | 219.0085;164.971;136.9988;124.9903;11 | (2S,3S)-2-(3,4,5-trihydroxyphenyl)-3,4-dihydro-2H-chromene-3,5,7-triol |
| 4 | 152.98888 | C_7_H_6_O_4_ | 90.9929;81.0116;53.0242 | Pyrocatechuic acid |
| 5 | 305.00513 | C_15_H _14_O_7_ | 219.007;166.9897;124.9903;57.0166 | (2S,3S)-2-(3,4,5-trihydroxyphenyl)-3,4-dihydro-2H-chromene-3,5,7-triol |
| 6 | 179.02145 | C_7_H_8_N_4_O_2_ | 163.9897;134.9709;122.002;94.0148;78.9942;56.7165 | Theophylline |
| 7 | 337.02539 | C_16_H_18_O_8_ | 191.0026;162.9957;119.0153;111.048 | (1R,3R,4S,5R)-1,3,4-trihydroxy-5-[(E)-3-(4-hydroxyphenyl)prop-2-enoyl]oxycyclohexane-1-car |
| 8 | 289.01294 | C_15_H_14_O_6_ | 245.0194;203.0175;178.9857;150.9982;109.0001;57.0189 | Epicatechin |
| 9 | 187.06079 | C_9_H_16_O_4_ | 125.0622;97.0378 | Azelaic acid |
| 10 | 337.02539 | C_16_H_18_O_8_ | 191.0026;162.9957;119.0153 | 3-p-Coumaroylquinic acid |
| 11 | 563.0306 | C_29_H_24_O_12_ | 563.0306 | Theaflavin |
|  |  |  | **Positive mode** |  |
| 12 | 181.0537 | C_7_H_8_N_4_O_2_ | 163.0478;138.0555;110.0645;83.0538;67.0241;56.0467 | Theobromine |
| 13 | 443.05554 | C_22_H_18_O_10_ | 291.0621;203.0842;139.0283;123.035 | Epicatechin gallate |
| 14 | 433.07153 | C_21_H_20_O_10_ | 397.063;367.0558;313.0449;283.0388 | 3-Genistein-8-C-glucoside |
| 15 | 433.07401 | C_21_H_20_O_10_ | 349.0584;337.04;313.0486;283.039 | 5,7-dihydroxy-2-(4-hydroxyphenyl)-6-[3,4,5-trihydroxy-6-(hydroxymethyl)oxan-2-yl]chromen |
| 16 | 617.09161 | C_27_H_30_O_15_ | 331.0691 | Kaempferol-3-O-rutinoside |
| 17 | 496.29129 | C_25_H_54_NO_6_P | 478.2919;313.2481;184.0589;104.0989 | 1-O-hexadecyl-2-C-methyl-3-phosphatidylcholine |
| 18 | 595.11206 | C_27_H_30_O_15_ | 287.0338;129.0464;71.0448 | Kaempferol-7-neohesperidoside |

**Table S.3 Summary of RNA-sequencing data**

| **sample** | **raw_reads** | **clean_reads** | **clean_bases** | **error_rate** | **Q20** | **Q30** | **GC_pct** |
| --- | --- | --- | --- | --- | --- | --- | --- |
| C1 | 131044420 | 124495912 | 9.34G | 0.02 | 98.19 | 94.55 | 50.23 |
| C2 | 131420468 | 127453272 | 9.56G | 0.02 | 98.35 | 94.9 | 50.94 |
| C3 | 141863036 | 133176168 | 9.99G | 0.02 | 98.39 | 95.05 | 51.39 |
| CD1 | 179882296 | 173654332 | 13.02G | 0.02 | 98.17 | 94.58 | 50.29 |
| CD2 | 148499048 | 142344044 | 10.68G | 0.02 | 98.36 | 95.03 | 50.7 |
| CD3 | 135512060 | 130385760 | 9.78G | 0.02 | 98.35 | 95 | 50.97 |
| TD1 | 118889332 | 113523104 | 8.51G | 0.02 | 98.38 | 95.07 | 50.57 |
| TD2 | 113331940 | 108984868 | 8.17G | 0.02 | 98.28 | 94.91 | 50.75 |
| TD3 | 124541744 | 118839076 | 8.91G | 0.02 | 98.14 | 94.51 | 50.41 |

Note: raw_ Reads: the number of reads in the raw data, clean_ Reads: the number of filtered reads of the raw data, clean_ Bases: base number of filtered raw data (clean base = clean reads * 150bp), error_ Rate: overall sequencing error rate of data, Q20: percentage of bases with phred value greater than 20 in total bases, Q30: percentage of bases with phred value greater than 30 in total bases, GC_ PCT: percentage of G and C in four bases of clean reads.
