# Supplementary figures and images for: Transcriptome and Gut Microbiota Profiling Revealed the Protective Effect of Tibetan Tea on Ulcerative Colitis in Mice
Source: Front Microbiol. 2022 Feb 14;12:748594. doi: 10.3389/fmicb.2021.748594 (PMC8882814; doi:10.3389/fmicb.2021.748594)

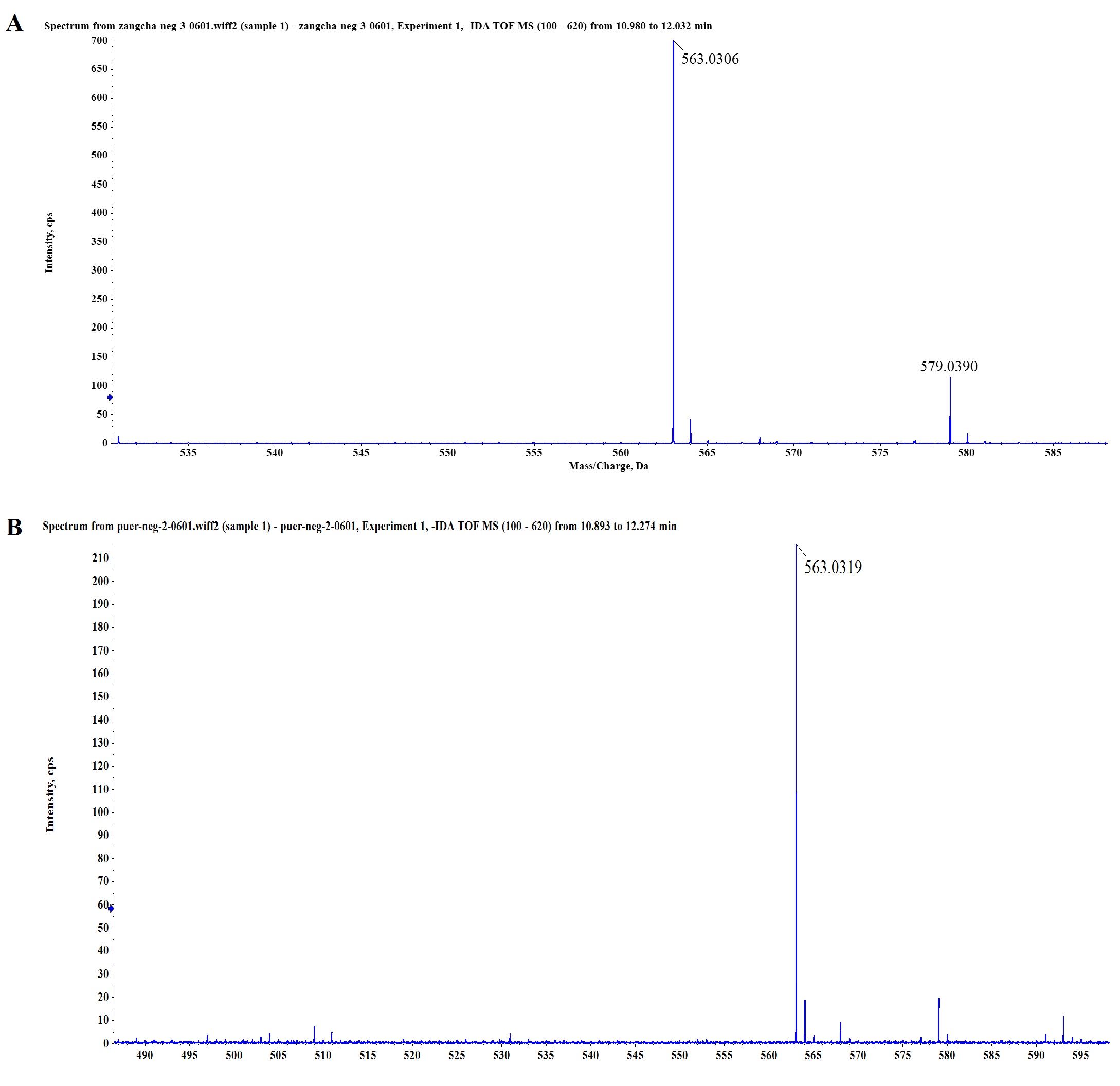

Supplement: Supplementary Figure 1 — Comparison of theaflavins content between Tibetan tea (A) and Pu-erh tea (B) by UHPLC-Q-TOF. [file Image_1.JPEG]

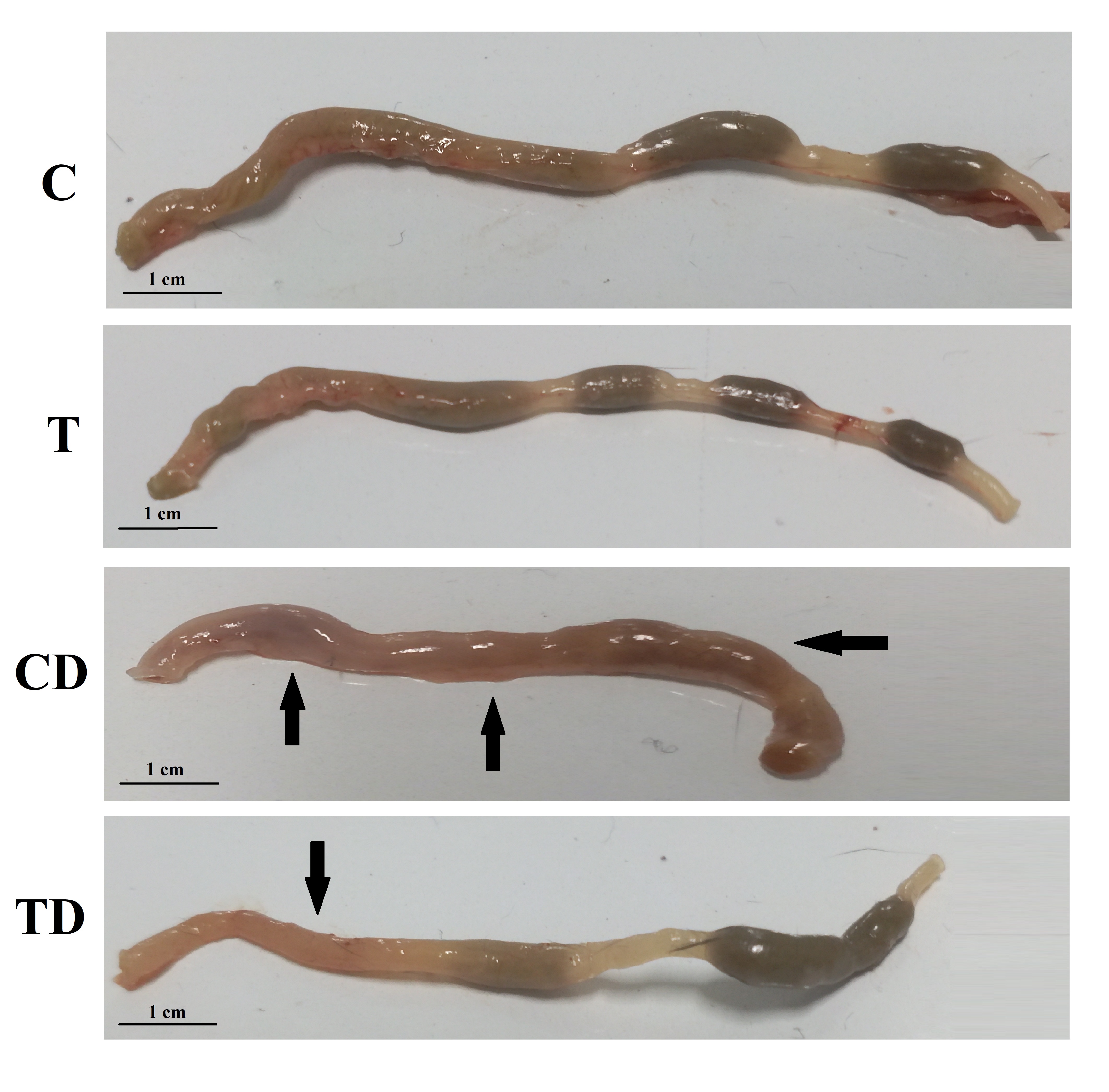

Supplement: Supplementary Figure 2 — Effects of TTE on colon length of mice after colitis induction. The arrow represents the site of colonic edema. Scale bars = 1 cm. [file Image_2.JPEG]
